# Phenotypic and genetic diversity in aposematic Malagasy poison frogs (genus *Mantella*)

**DOI:** 10.1002/ece3.4943

**Published:** 2019-02-05

**Authors:** Karina Klonoski, Ke Bi, Erica Bree Rosenblum

**Affiliations:** ^1^ Department of Environmental Science, Policy, and Management University of California, Berkeley Berkeley California; ^2^ Museum of Vertebrate Zoology University of California, Berkeley Berkeley California; ^3^ Computational Genomics Resource Laboratory (CGRL), California Institute for Quantitative Biosciences (QB3) University of California, Berkeley Berkeley California

**Keywords:** color evolution, hybridization, *Mantella*, poison frogs, polymorphism, population genomics

## Abstract

Intraspecific color variation has long fascinated evolutionary biologists. In species with bright warning coloration, phenotypic diversity is particularly compelling because many factors, including natural and sexual selection, contribute to intraspecific variation. To better understand the causes of dramatic phenotypic variation in Malagasy poison frogs, we quantified genetic structure and color and pattern variation across three closely related species, *Mantella aurantiaca*, *Mantella crocea*, and *Mantella milotympanum*. Although our restriction site‐associated DNA (RAD) sequencing approach identified clear genetic clusters, they do not align with current species designations, which has important conservation implications for these imperiled frogs. Moreover, our results suggest that levels of intraspecific color variation within this group have been overestimated, while species diversity has been underestimated. Within major genetic clusters, we observed distinct patterns of variation including: populations that are phenotypically similar yet genetically distinct, populations where phenotypic and genetic breaks coincide, and populations that are genetically similar but have high levels of within‐population phenotypic variation. We also detected admixture between two of the major genetic clusters. Our study suggests that several mechanisms—including hybridization, selection, and drift—are contributing to phenotypic diversity. Ultimately, our work underscores the need for a reevaluation of how polymorphic and polytypic populations and species are classified, especially in aposematic organisms.

## 1 INTRODUCTION

The origin and maintenance of intraspecific color variation and its consequences for speciation have long captivated evolutionary biologists (e.g., Huxley, 1955; Endler, 1980; Gray & McKinnon, 2007; Corl, Davis, Kuchta, & Sinervo, 2010, Hugall & Stuart‐Fox, 2012). Examples of color variation in nature are widespread and have been documented across many diverse taxonomic groups, including reptiles, fish, mammals, birds, and invertebrates (e.g., Sandoval, 1994; Sinervo, Bleay, & Adamopoulou, 2001; Galeotti, Rubolini, Dunn, & Fasola, 2003; Hoekstra, Drumm, & Nachman, 2004; Maan et al., 2008). Previous work has indicated that color variation observed within or between populations may be mediated by natural selection (e.g., apostatic selection, divergent selection in different substrate or light environments as in Sandoval, 1994; Hoekstra et al., 2004), sexual selection (e.g., mate choice, variation in male mating strategies as in Sinervo & Lively, 1996; Kingston, Rosenthal, & Ryan, 2003), genetic drift (Hoffman, Schueler, Jones, & Blouin, 2006), or some combination of these factors (e.g., Endler, 1991; Oxford, 2005; Reynolds & Fitzpatrick, 2007). Intraspecific color variation can also potentially give rise to new species, especially when natural or sexual selection reduces gene flow between alternative morphs (e.g., Rosenblum & Harmon, 2011).

Examples of color variation in aposematic organisms—where conspicuous warning signals advertise toxicity or unpalatability to predators (Poulton, 1890)—are particularly compelling. Aposematic colors are often highly contrasting, variable, and potentially exhibit trade‐offs between natural and sexual selection (Crothers & Cummings, 2013; Estrada & Jiggins, 2008; Jiggins, Naisbit, Coe, & Mallet, 2001; Nokelainen, Hegna, Reudler, Lindstedt, & Mappes, 2012; Reynolds & Fitzpatrick, 2007; Stevens & Ruxton, 2012; Summers, Symula, Clough, & Cronin, 1999). Historically, variation in aposematic signals has been considered perplexing from a theoretical perspective because phenotypic diversity is expected to be highly constrained in such systems due to positive frequency‐dependent selection via predation (Briolat et al., 2018; Endler & Greenwood, 1988; Mallet & Joron, 1999). Once a predator has learned to associate toxicity with a particular phenotype, protection should be conferred to those organisms displaying a similar phenotype, encouraging uniformity in warning coloration. According to theoretical predictions of predator avoidance learning, novel phenotypes should be unrecognizable to predators as toxic and thus quickly removed from populations (Guilford & Dawkins, 1993; Mallet & Barton, 1989; Müller, 1879). In recent years, however, studies have identified many biotic and abiotic factors—including intraspecific communication, parasite load, temperature and variability in predator learning and sensory abilities—that contribute to variation in aposematic signals (reviewed in Briolat et al., 2018). Growing awareness of the variety of selective factors influencing aposematic coloration has led scientists to encourage a more holistic approach when investigating diversity in warning coloration and to consider the range of factors that may be at play in maintaining phenotypic variation (Briolat et al., 2018).

Studies of color variation within aposematic species have traditionally focused on systems demonstrating either multiple color morphs within a population (i.e., polymorphism) or geographic color variation among populations (i.e., polytypism) (reviewed in Briolat et al., 2018). In systems with high color variability, however, determining whether color variants represent different species, different populations, or different morphs within populations is difficult particularly when genetic structure is not well resolved. Distinguishing between species, populations, and morphs can be especially challenging in phenotypically diverse poison frog groups, where high rates of phenotypic variation can confound our understanding of species delimitations (Posso‐Terranova & Andrés, 2018; Roland et al., 2017; Tarvin, Powell, Santos, Ron, & Cannatella, 2017).

In many instances, the inability to distinguish whether color variation occurs within populations, between populations, or between species is further compounded by nonexistent or limited genetic datasets, which lack the resolution needed to clarify relationships among populations. Tarvin et al. (2017) recently demonstrated that the level of interspecific mitochondrial divergence among four distinct poison frog species was comparable to the divergence levels observed between populations considered to be a single polymorphic species (Hauswaldt, Ludewig, Vences, & Pröhl, 2011). Noting the limitations of mitochondrial data in resolving species boundaries, the authors explicitly called for genome‐level studies, in combination with information on phenotypic diversity and natural history, to understand relationships in these complicated systems (Tarvin et al., 2017). The power of more comprehensive genetic datasets to resolve genetic structure in poison frogs has been demonstrated in recent studies where Neotropical poison frogs considered to be a single species in fact contained multiple genetic lineages that potentially represent new species (Posso‐Terranova & Andrés, 2018; Roland et al., 2017).

While the relationship between phenotypic and genetic diversity has been extensively studied in the Neotropical poison frogs (e.g., Wang & Summers, 2010; Twomey et al., 2013; Roland et al., 2017; Tarvin et al., 2017), there is an entirely separate radiation of poison frogs in which color diversity has never been examined. Endemic to Madagascar, the *Mantella* genus describes sixteen species of toxic, diurnal frogs exhibiting variable coloration and pattern both within and among species (Glaw & Vences, 2007). Commonly called Malagasy poison frogs, the bright coloration displayed by many species within this group is presumed to be aposematic. The toxic skin alkaloids found in *Mantella* species are believed to be derived from arthropod prey (Daly, Andriamaharavo, Andriantsiferana, & Myers, 1996; Daly, Garraffo, Hall, & Cover, 1997), similar to the Neotropical poison frogs, and variation in alkaloid composition has been observed among species, populations, and habitats (Andriamaharavo et al., 2015; Daly et al., 2008). The mechanism of chemical defense is hypothesized to have evolved convergently in Neotropical and Malagasy poison frogs (Clark, Raxworthy, Rakotomalala, Sierwald, & Fisher, 2005). Despite their high degree of phenotypic variation and apparent similarity to the Neotropical poison frogs (Heying, 2001b; Rojas, 2016), little is known about the natural history, ecology, and genetic background of Malagasy poison frogs.

Within the *Mantella* genus, one complex of three closely related species, *Mantella aurantiaca*, *Mantella crocea*, and *Mantella milotympanum*, demonstrates a particularly high degree of variability in conspicuous coloration and pattern. Found in the rainforests of central‐eastern Madagascar, the geographic range of all three species is highly restricted and patchy in its distribution (Bora et al., 2008). Among and within populations, there is exceptional phenotypic variation both in dorsal coloration, which ranges from red to green at the extremes, and in patterning elements, which are variable in the degree of ventral spotting and black banding present on the side. Yet, any attempt to understand the phenotypic diversity in this group is hindered by the unresolved taxonomy of these species. Previous genetic work is limited to a handful of mitochondrial DNA and allozyme studies, which have yielded somewhat confusing results (Chiari et al., 2004; Schaefer, Vences, & Veith, 2002; Vences, Chiari, Raharivololoniaina, & Meyer, 2004; Vences, Hille, & Glaw, 1998). *Mantella aurantiaca*, *M. milotympanum*, and *M. crocea* are thought to fall within the *Mantella madagascariensis* group, one of five clades within the *Mantella* genus, though their position in this group is controversial (Schaefer et al., 2002; Vences et al., 2004). Population genetic studies have detected high degrees of haplotype sharing between *M. milotympanum* and *M. crocea*, resulting in the hypothesis that these two species are conspecific (Chiari et al., 2004; Vences et al., 2004). Additionally, frog populations displaying patterning that is intermediate between that of *M. crocea* and *M. milotympanum* exist in the wild and are referred to as *M. cf. milotympanum *in the literature (Chiari et al., 2004). Evidence of haplotype sharing between *M. aurantiaca* and *M. crocea* (Chiari et al., 2004; Vences et al., 2004) has also prevented taxonomic resolution within this group, and species designations remain controversial.

Given the lack of resolution in previous molecular studies, it is apparent that a high‐resolution genetic dataset is needed to both clarify relationships within this group and to determine whether observed color variants represent distinct species or morphs. In this study, we used a restriction site‐associated (RAD) sequencing approach, in combination with multiple matrix regression analysis, to compare variation in dorsal coloration and side and ventral patterning with genetic and geographic distance across the entire known range of three species of Malagasy poison frog. Specifically, our objectives were to (a) clarify genetic structure among populations of *M. crocea*, *M. milotympanum,* and *M. aurantiaca*, (b) quantify variation in dorsal coloration and side and ventral patterning both among and within populations, and (c) describe the relationship between genetic diversity, phenotypic diversity, and geographic distance for all major genetic clusters within this three‐species complex. This study—the first quantitative and objective exploration of color diversity in the *Mantella* genus—not only provides a foundation for future studies of color evolution in Malagasy poison frogs but also identifies several critical issues that should be more thoroughly considered in any investigation of aposematic organisms presumed to be polymorphic or polytypic.

## 2 MATERIALS AND METHODS

### 2.1 Field sampling

**Figure 1:**
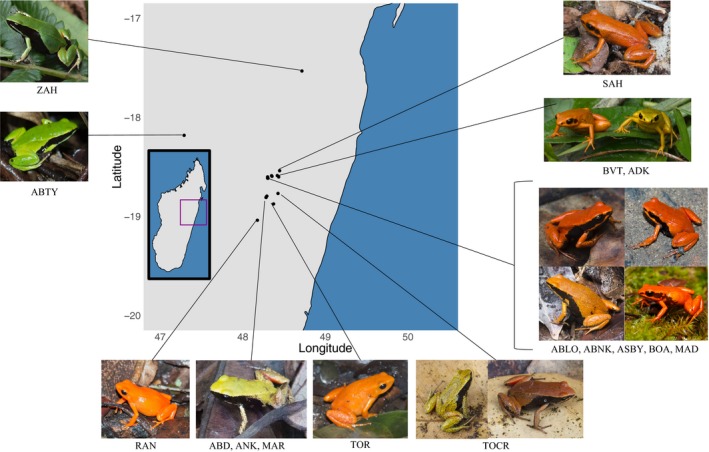
Sampling localities for 16 populations of *Mantella crocea*, *Mantella aurantiaca*, and *Mantella milotympanum* across central‐eastern Madagascar. Representative individuals from a population or group of populations are pictured next to population labels. Images shown here do not represent the entire range of observed phenotypic diversity

We sampled three closely related species, currently named *M. aurantiaca*, *M. crocea*, and *M. milotympanum*, throughout their entire known range in central‐eastern Madagascar (Figure 1). We also sampled individuals containing an intermediate phenotype between *M. crocea* and *M. milotympanum*, hereafter referred to as *M. cf. milotympanum*, following previous nomenclature in the literature. Overall, we sampled 88 frogs from 16 populations. Fieldwork was conducted during the rainy breeding season over 3 years: January–February 2014, January–February 2015, and November 2015–January 2016. We captured the frogs by hand and transported them back to a field laboratory where all data collection occurred. We collected digital photographs and toe clips from 11 *M. aurantiaca* individuals from two populations, 34 *M. crocea* individuals from six populations, 19 *M. milotympanum* individuals from three populations, and 24 *M. cf. milotympanum* individuals from five populations. Several studies have demonstrated that toe‐clipping has no significant impact on frog survival, body condition, or growth (reviewed in Perry, Wallace, Perry, Curzer, & Muhlberger, 2011). Toe clips were preserved in salt‐saturated DMSO and stored at room temperature. All data were collected on the same day that frogs were captured. Frogs were held overnight and released to their site of capture the following morning. All animal handling procedures were approved by the Animal Care and Use Committee at the University of California at Berkeley (AUP‐2015‐01‐7083). Collection and exportation of samples were performed under permits issued by the Direction Générale des Forêts, Direction de la Conservation de la Biodiversité et du Système des Aires Protégées, and Ministere de l’Environment, de l’Ecologie et des Forêts in Madagascar (collection permits: 315/13/MEF/SG/DGF/DCB.SAP/SCB, 335/14/MEF/SG/DGF/DCB.SAP/SCB, and 336/14/MEF/SG/DGF/DCB.SAP/SCB; export permits: 051C‐EA02/MG14, 048C‐EA02/MG15, and 002C‐EA01/MG16).

### 2.2 Quantification of phenotypic variation

To characterize frog coloration and pattern, we photographed the dorsal, ventral, and side surfaces of all frogs in a standardized manner following a protocol modified from Stevens, Párraga, Cuthill, Partridge, and Troscianko (2007). Photographs of all frogs were taken after transportation to the field laboratory, but before any other handling occurred. Frogs were always photographed in natural light during the hours of 1:00–5:00 p.m. using a Pentax K‐30 digital single‐lens reflex camera fitted with a Pentax 18–135 mm lens. All frogs were photographed on a white paper background with a scale bar and a white–gray–black standard present (QPcard 101; gray standard reflectance value of 18%). Two individuals (ABNK09 and RAN11) were excluded from our phenotypic analysis due to unsuitable photographs.

To quantify dorsal coloration, we first used the Image Calibration and Analysis Toolbox (Troscianko & Stevens, 2015), utilized within ImageJ v1.51 (Schneider, Rasband, & Eliceiri, 2012), to generate aligned and normalized images from our RAW photographs, which enabled us to objectively measure color and pattern. After standardization, we selected two regions of interest on the frog’s dorsal surface in which to quantify color: one 3 × 3 mm square toward the back of the frog dorsum, and one 3 × 1 mm rectangle behind the frog’s right arm. Regions of interest on the frog’s dorsum were manually selected in order to avoid any glare present in photographs as well as any injuries on the frog’s dorsal surface that resulted in discoloration. After selecting regions of interest, we used the Batch Image Analysis function of the toolbox to extract the red color values, green color values, and blue color values (hereafter referred to as RGB values) for each frog. R values, G values, and B values were averaged for all pixels within the regions of interest. Color values were averaged separately for each color channel (R, G, and B).

To transform RGB values into measurements relevant to a vertebrate visual system, we followed the protocol of Krohn and Rosenblum (2016), modified from Endler (2012) and McKay (2013). Briefly, we calculated three axes from our RGB values corresponding to a red‐green channel, (R − G)/(R + G), a blue‐green channel, (G − B)/(G + B), and a luminance channel, which we defined as untransformed R values. Because luminance is processed separately in vertebrates (Endler, 2012; Endler & Mielke, 2005), we used our other two axes, (R − G)/(R + G) and (G − B)/(G + B), to plot dorsal coloration as a point in a two‐dimensional color space based on a red‐green channel and a blue‐green channel. In our two‐dimensional color space, (R − G)/(R + G) represented the *x*‐axis and (G − B)/(G + B) represented the *y*‐axis. From this color space, we calculated chroma and hue values following Krohn and Rosenblum (2016). Chroma was calculated as the hypotenuse of the triangle formed by the x and y axes, and hue was calculated as the angle between the hypotenuse and the *x*‐axis. We used these values of chroma, hue, and luminance to characterize dorsal coloration.

To quantify side and ventral pattern, we again used the Image Calibration and Analysis Toolbox (Troscianko & Stevens, 2015) to generate aligned and normalized images from RAW photographs. Next, we manually outlined the ventral and side surfaces of frogs on the standardized images using the polygon and brush selection tools. We selected the entire ventral and side surfaces to obtain a comprehensive measure of overall pattern on each surface. After manually selecting the regions of interest, we used the scale bars present in each photograph to scale all images to the same number of pixels per millimeter (side surfaces = 19 px/mm; ventral surfaces = 18.6 px/mm), which is necessary for pattern analysis. We performed a granularity analysis, which is based on Fast Fourier bandpass filtering, using the Image Calibration and Analysis Toolbox implemented in ImageJ. For our side pattern analysis, we specified Fast Fourier Transform Bandpass filters at 16 levels, starting at two pixels and increasing by a multiple of the square root of two until 430 pixels. For our ventral pattern analysis, we specified Fast Fourier Transform Bandpass filters at 14 levels, starting at two pixels and increasing by a multiple of the square root of two until 193 pixels. From our granularity analysis, we generated descriptive summary statistics to estimate pattern contrast, pattern diversity, and luminance contrast for both side and ventral pattern. Granularity analysis has been increasingly used to measure pattern in a variety of organisms (e.g., Barbosa et al., 2008; Stoddard & Stevens, 2010) and draws on basic characteristics of early‐stage visual processing present across diverse animals (Campbell & Rodson, 1968; Godfrey, Lythgoe, & Rumball, 1987; Pérez‐Rodríguez, Jovani, & Stevens, 2017; Stoddard & Stevens, 2010). Conversely, color adjacency analysis, a method which has previously been used to quantify pattern in poison frogs, does not represent visual processing of pattern (Pérez‐Rodríguez et al., 2017).

Our dataset included both males and females (58 males, 26 females, two juveniles). Preliminary analyses did not reveal an effect of sex on frog coloration or pattern, so sexes were lumped for the analyses presented here. However, our study design did not explicitly aim to quantify sexual dimorphism, and future studies can investigate this question with targeted sampling.

### 2.3 Quantification of genetic variation

We extracted DNA from toe clips using Qiagen DNeasy extraction kits (Qiagen, Valencia, CA, USA) generally following the manufacturer’s protocol with two modifications: 4 µl of 1 mg/ml RNase A was added to each sample after lysis, and DNA was eluted in 45 µl of 1× LTE buffer to maximize concentration. Prior to library preparation, we checked the quality of extracted DNA using agarose gels and quantified DNA using a Qubit 2.0 Fluorometer (ThermoFisher, Waltham, MA, USA).

We constructed a restriction site‐associated DNA (RAD) library following the protocol of Ali et al. (2016), without the targeted bait capture step, otherwise referred to as the “bestRAD” protocol. During preparation of our bestRAD library, we digested 50 ng of DNA from each individual with SbfI‐HF (New England Biolabs, Ipswich, MA, USA) restriction enzyme, performed size selection with magnetic beads, and amplified our library using 12 cycles of polymerase chain reaction (PCR). We sequenced our library on two lanes of the Illumina HiSeq 4000 at the U.C. Davis Genome Center with 150 bp paired‐end reads.

### 2.4 RADseq data processing

To process RADseq data, we used pipelines implemented in a customized PERL workflow that also utilized various external programs (pipelines can be accessed through https://github.com/CGRL-QB3-UCBerkeley/RAD). We first demultiplexed raw fastq reads using internal barcode sequences and allowing for one mismatch in barcode sequence. We removed demultiplexed reads that did not include the expected restriction enzyme cut site at the beginning of the read, again allowing for one mismatch in cut site sequence, and also removed exact duplicates using Super Deduper (https://github.com/dstreett/Super-Deduper). To filter reads, we used Cutadapt (Martin, 2011) and Trimmomatic (Bolger, Lohse, & Usadel, 2014) to trim adapter contamination and low quality reads. We removed filtered reads that were shorter than 50 bp. After cleaning and filtering reads, we used cd‐hit (Fu, Niu, Zhu, Wu, & Li, 2012; Li & Godzik, 2006) to cluster forward reads of each individual at 95% similarity, retaining only those clusters with at least two supported reads. For each cluster, we used the sequence identified as representative by cd‐hit as our marker. Retained markers were next masked for repetitive elements, low complexities, and short repeats with Ns using RepeatMasker (Smit, Hubley, & Green, 2014) with “frog” as a database. Post‐masking, we removed markers where more than 30% of nucleotides were Ns. To remove potential paralogs present within each individual, we used Blastn (Altschul, Gish, Miller, Myers, & Lipman, 1990) to compare all clustered loci against themselves, and subsequently eliminated any locus that matched a locus other than itself. Next, remaining RAD markers from each individual were combined and clustered for all individuals using cd‐hit. We used a similarity threshold of 90% to select for markers containing at least 50 nucleotides and shared by at least 60% of all individuals. This served as our reference genome for all samples, and we subsequently aligned each individual’s cleaned sequence reads to this reference using Novoalign (http://www.novocraft.com). We only retained those reads that mapped uniquely to the reference. Using Picard (http://www.picard.sourceforge.net) and GATK (McKenna et al., 2010), we added read groups and performed realignment around indels. To generate quality control information in VCF format, we used SAMtools/BCFtools (Li et al., 2009), after which data were further filtered using a custom method, SNPcleaner (https://github.com/tplinderoth/ngsQC/tree/master/snpCleaner; Bi et al., 2013), that was slightly modified to remove sites around indels and implemented in our pipelines. Additionally, we filtered out markers where more than two alleles were called at any site, and also masked sites that were within 10 bp of any indel. Only individual sites falling within the first to ninety‐ninth percentile of overall coverage among all samples were retained. We also removed SNPs failing to pass a one‐tailed HWE exact test (1e−4) or showing strong base quality bias (1e−100). To avoid bias resulting from excessive missing data, we only used sites present in at least 60% of individuals with at least 3× coverage in our downstream analyses.

### 2.5 Population genomic analyses

We used genotype likelihoods instead of genotype calls whenever possible to account for uncertainty in our data. Because our data showed low to medium coverage (1.8–13.9×, with an average of 5.4×), SNP and genotype calls based on allele counts could potentially cause bias or introduce noise in downstream analyses (Johnson & Slatkin, 2008; Lynch, 2008). Genotype likelihoods were calculated in an empirical Bayesian framework using ANGSD (http://www.popgen.dk/angsd/index.php/ANGSD; Korneliussen, Albrechtsen, & Nielsen, 2014), a software that is specialized for analyzing low to medium coverage next‐generation sequencing data. The majority of downstream analyses conducted in ANGSD are performed based on likelihood of site allele frequencies, genotype likelihoods, or genotype posterior probabilities. For analyses that required SNP or genotype calling, we only included high‐confidence variants (identified using a likelihood ratio test to determine variable sites with *p*‐values <1e−6 and genotype posterior probabilities >0.95) where at least 80% of individuals showed at least 3× coverage.

To characterize population structure for all samples, we first performed a Principal Components Analysis (PCA) of the covariance matrix of posterior genotype probabilities implemented in ngsTools (http://github.com/mfumagalli/ngsTools; Fumagalli, Vieira, Linderoth, & Nielsen, 2014). Next, we used a neighbor‐joining network (NeighborNet) analysis based on uncorrelated *p*‐distances in Splitstree (Huson, 1998; Huson & Bryant, 2006) to visualize population structure. We adhered to the stringent thresholds mentioned above to call a set of high‐quality variants, which were used to compute a genetic distance matrix for Splitstree using the Adegenet package in R (Jombart, 2008). We also quantified the population structure of all samples using NGSadmix (Skotte, Korneliussen, & Albrechtsen, 2013), which relies on genotype likelihoods. Because there were sixteen populations present in our study, we estimated individual admixture proportions with the number of clusters ranging from one to seventeen (*K* = 1–17), with ten replicates per *K* value. We then used the Evanno method (Evanno, Regnaut, & Goudet, 2005) to identify the most likely *K* value.

To characterize fine‐scale population structure, we performed an NGSadmix analysis within each major genetic cluster. We again estimated individual admixture proportions with the number of clusters ranging from one to one more than the total number of populations (*K* = 1–3 for Cluster A; *K* = 1–7 for Cluster B; *K* = 1–9 for Cluster C; *K* = 1–15 for candidate hybrid populations). We ran NGSadmix with ten replicates for each *K* value and used the Evanno method to determine the most likely *K* value. We assigned admixed populations to the NGSadmix group from which more than 50% of its admixture was drawn. Finally, we calculated *F*_ST_ values for all possible pairwise population comparisons using the realSFS function of ANGSD.

### 2.6 Integration of genomic and phenotypic datasets

We used Mantel and partial Mantel tests to investigate the relationship between genetic, geographic, and phenotypic distance for each major cluster in our study. Some concerns have been raised over the use of Mantel tests in population genetics, especially in regards to inflated type I error rates for partial Mantel tests when spatial autocorrelation is present (Diniz‐Filho et al., 2013; Guillot & Rousset, 2013; Legendre & Fortin, 2010). Therefore, we rely most heavily on pairwise comparisons, and we are cautious in our interpretation of partial Mantel results. In addition, we focus on comparisons across major genetic groupings, where any potential biases should be comparable. Although we are conservative throughout about linking pattern to process, our results highlight complexes with interesting spatial patterns of genetic and phenotypic variation.

Our regression analysis was performed on 86 individuals and excluded the two samples indicated above. To generate our geographic distance matrix, we used the Geographic Distance Matrix Generator (Ersts, 2018). To generate our genetic distance matrix, we used ngsDist (Vieira, Lassalle, Korneliussen, & Fumagalli, 2016) to estimate pairwise genetic distances using genotype posterior probabilities. To quantify phenotypic distances among individuals, we generated three separate distance matrices: a dorsal coloration distance matrix, a side pattern distance matrix, and a ventral pattern distance matrix. To generate our dorsal coloration distance matrix, we first standardized digital photographs, extracted and averaged RGB values from regions of interest, and transformed RGB values to a two‐dimensional color space as described above. We then calculated the Euclidean distance between points in this conceptual color space to generate measures of pairwise distance in dorsal coloration. To generate our side and ventral pattern distance matrices, we used differences in luminance (with R as the luminance channel) to characterize the pairwise distances in pattern among individuals. We designated R as the luminance channel because many vertebrates are believed to use long‐wavelength sensitive (LWS) cones to detect achromatic signals (Endler & Mielke, 2005; Osorio & Vorobyev, 2005). After standardizing digital photographs, selecting regions of interest, and scaling all pictures as described above, we calculated the number of pixels that fell into each of 95 luminance bins ranging from 0% to 100% reflectance for each frog’s surfaces separately (ventral and side). Next, we used the toolbox’s Luminance Distribution Difference Calculator to compare the luminance distribution histograms among each pair of frogs and to generate pairwise measures of difference in luminance distribution, which we used as our measure of variation in ventral and side pattern. This methodology follows the recommendation of the toolbox’s user guide because pattern differences in this study system are characterized by discrete patches of high and low luminance values. All Mantel and partial Mantel tests were performed in R version 3.4.3 using the vegan package (R Core Team, 2017), and each test used Pearson’s method of correlation and performed 999 permutations.

## 3 RESULTS

Our sequencing efforts yielded a high‐quality dataset. After filtering reads for intact barcode sequences and restriction enzyme sites, the number of reads per sample ranged from 0.86 to 13.8 million, with an average of 4.48 million reads per sample. After self‐blasting, we obtained an average of 43,559 RAD loci per individual, with the number of loci ranging from 15,491 to 59,488 per sample. The final pseudo‐reference genome, which was generated by clustering loci across all samples and included those loci shared by at least 60% of the samples, contained 35,113 loci. The average coverage per individual was 5.4× and ranged from 1.8× to 13.9×. After raw variant filtering, we retained 2,284,942 sites derived from 21,733 loci for ANGSD analyses. Above 65% of the samples in our dataset have at least 3× coverage at these loci.

**Figure 2:**
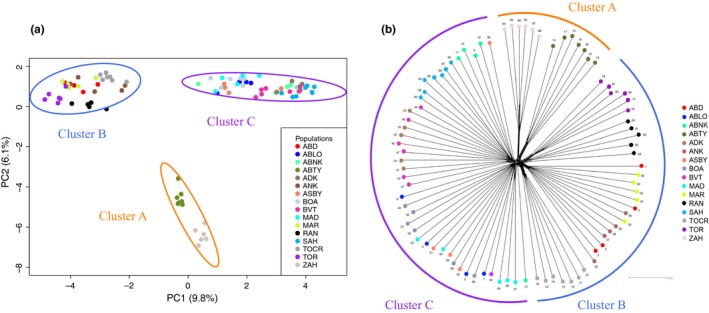
Patterns of genetic variation among individuals from all sampling localities. Plots show PCA (panel a) and Splitstree diagram (panel b) for all individuals, with colors denoting sampling localities. Ellipses on the PCA plot represent the 95% confidence intervals around the mean of each group identified in our NGSadmix analysis (Clusters A, B, and C). Semi‐circles on Splitstree diagram denote groups identified in NGSadmix analysis and again correspond to Clusters A, B, and C. NeighborNet analysis in panel (b) was based on *p*‐distances in Splitstree

**Figure 3:**
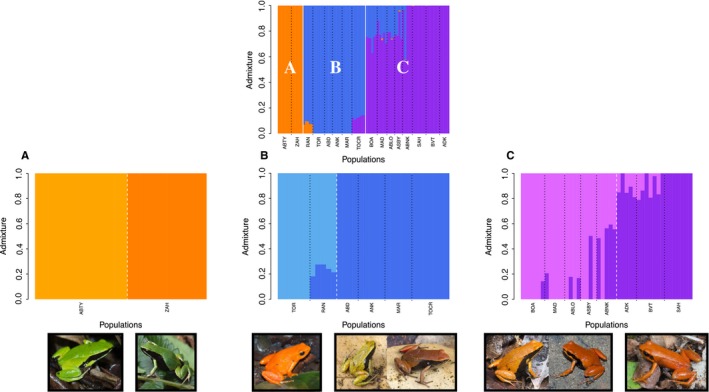
Population structure determined by NGSadmix analysis. The top plot shows NGSadmix clustering for *K* = 3 (the most likely *K*) when all samples are included. Dashed black lines separate geographic sampling locations, while solid white lines separate groups identified in NGSadmix analysis (designated here as Clusters A, B, and C). NGSadmix sub‐plots show fine‐scale population structure within each major genetic cluster (A, B, and C) for *K* = 2 (determined to be the most likely *K* for each cluster). Within each sub‐plot, black dashed lines separate geographic sampling locations, while dashed white lines separate groups identified in NGSadmix analysis. Below each sub‐plot are pictures of representative individuals from the population or populations contained within each NGSadmix group

**Figure 4:**
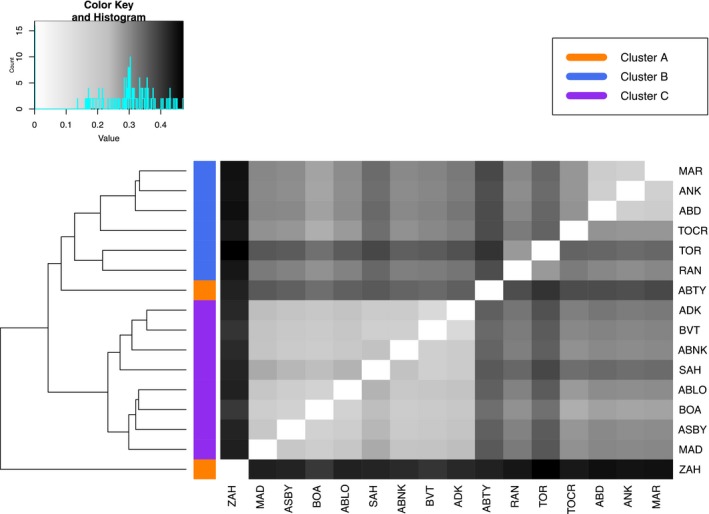
Dendrogram and heat map of pairwise *F*_st_ values for all sampling localities. Lighter cells denote lower pairwise *F*_st_ values (less genetic differentiation), while darker cells denote higher pairwise *F*_st_ values (more genetic differentiation)

We found that *M. aurantiaca*, *M. crocea*, and *M. milotympanum* populations were highly structured with three distinct groups emerging. Our PCA, based on 2,284,942 sites, revealed three main clusters: Cluster A contained the two most geographically remote *M. crocea* populations, Cluster B contained the remaining *M. crocea* populations and the two *M. aurantiaca* populations, and Cluster C contained all *M. milotympanum* and all *M. cf. milotympanum* populations (Figure 2a). These three clusters were supported by our NGSadmix analysis, which also identified three groups (*K* = 3 based on Evanno et al., 2005 method) corresponding to the same population groupings (Figure 3). Our splitsTree analysis, based on 14,367 high‐quality SNPs, further supported this general pattern of genetic variation partitioning (Figure 2b), though there were notable differences in the degree of genetic admixture present within clusters. The admixture detected in our NGSadmix and splitsTree analysis was concordant with our pairwise population *F*_ST_ value comparisons, which were lower among admixed populations (Figure 4). Still, *F*_ST_ values were relatively high for all pairwise population comparisons, including those that were admixed. It is important to note that geographic and genetic distance were not equivalent across major genetic clusters. While populations within some clusters were both geographically and genetically disparate (as in Cluster A), other populations were geographically more proximate with varying degrees of genetic distance between them (as in Clusters B and C).

**Figure 5:**
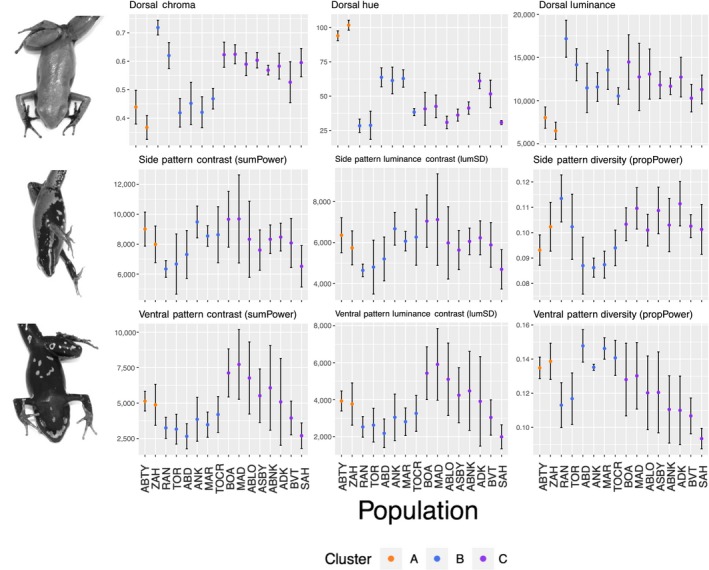
Variation in dorsal coloration (chroma, hue, and luminance) and side and ventral pattern (pattern contrast, luminance contrast, and pattern diversity) across all sampling localities. Mean values and standard deviation for each population are depicted in the graphs

Within Cluster A, both our PCA and NGSadmix analysis indicated that each population was a genetically distinct entity. Our NGSadmix analysis identified two groups (*K* = 2 based on Evanno et al., 2005 method) corresponding to each population (Figure 3). These populations were also clearly differentiated in our splitsTree analysis, with no admixture occurring between them (Figure 2b). In fact, the pairwise *F*_ST_ value between these two populations (0.43) was among the highest in our dataset (Figure 4). Within this cluster, Mantel and partial Mantel tests confirmed that genetic distance was most highly correlated with geographic distance (*r* = 0.9444; *p*‐value = 0.001; Table 1). Although dorsal coloration and side pattern were also correlated with genetic variation, neither phenotypic trait remained significantly associated with genetic distance after accounting for the effect of geographic distance in partial Mantel tests (side pattern: *r* = −0.1835; *p*‐value = 0.994; dorsal coloration: *r* = 0.1033, *p*‐value = 0.129). Additionally, the degree of phenotypic variability was much lower in this cluster than in the others, as side pattern was the only phenotypic trait that was significantly correlated with geographic distance when accounting for genetic distance (*r* = 0.3006; *p*‐value = 0.008). Phenotypically, populations within this cluster were relatively uniform, and there was little variation in either dorsal coloration or in side and ventral pattern (Figure 5).

**Table 1:** Matrix regression results of variables that correlate with genetic distance within major genetic clusters

Group	X matrix	Mantel *r*	*p*
Cluster A	Dorsal coloration	0.3089	0.019*
	Geographic dist.	0.9444	0.001* ^,^ †
	Side pattern	0.3066	0.006*
	Ventral pattern	0.0529	0.258
Cluster B	Dorsal coloration	0.3715	0.001* ^,^ †
	Geographic dist.	0.3414	0.011*
	Side pattern	0.2880	0.002*
	Ventral pattern	0.3467	0.001*
Cluster C	Dorsal coloration	−0.0202	0.537
	Geographic dist.	0.1038	0.018* ^,^ †
	Side pattern	−0.1280	0.897
	Ventral pattern	−0.0956	0.876
Candidate hybrids	Dorsal coloration	−0.0012	0.472
	Geographic dist.	0.1391	0.050* ^,^ †
	Side pattern	−0.0386	0.740
	Ventral pattern	0.0471	0.153

Notes

“Candidate hybrids” refer to the subset of samples that includes admixed individuals and the clusters from which this admixture was drawn (Clusters B and C).

* Significant *p*‐values.

† The variable with the highest Mantel *r* statistic.

Within Cluster B, we also found evidence for two distinct groups based on our PCA and NGSadmix analysis. Our NGSadmix analysis identified two groups (*K* = 2 based on Evanno et al., 2005 method), one containing the two *M. aurantiaca *populations and one containing the four remaining *M. crocea* populations (Figure 3), though there was some admixture between one *M. crocea* and one *M. aurantiaca* population. Our splitsTree analysis supported this general pattern and also showed admixture among the three geographically closest *M. crocea* populations (Figure 2b). Pairwise *F*_ST_ values among these three admixed *M. crocea* populations ranged from 0.17 to 0.18, which is low in comparison to the range of values in our dataset (Figure 4). The *F*_ST _values between any of these three admixed *M. crocea* populations and the fourth remaining *M. crocea* population ranged from 0.28 to 0.29 (Figure 4). Comparatively, values between either *M. aurantiaca* population or *M. crocea* populations ranged from 0.30 to 0.35 (Figure 4). Within this group, Mantel and partial Mantel tests confirmed that genetic distance was most highly correlated with variation in dorsal coloration (*r* = 0.3715; *p*‐value = 0.001; Table 1). Even after accounting for the effects of geographic distance in a partial Mantel test, dorsal coloration remained significantly associated with genetic variation (*r* = 0.2398; *p*‐value = 0.002). Variation in ventral patterning also remained significantly correlated with genetic distance after controlling for geographic distance (*r* = 0.2152; *p*‐value = 0.011). All three quantified phenotypic traits were significantly correlated with geography after controlling for genetic distance (dorsal coloration: *r* = 0.4589; *p*‐value = 0.001; side pattern: *r* = 0.5174; *p*‐value = 0.001; ventral pattern: *r* = 0.437; *p*‐value = 0.001). Phenotypic discrepancies between the *M. crocea* and *M. aurantiaca* groups were most evident in dorsal coloration, particularly in dorsal chroma and hue (Figure 5). Although genetic distance was correlated with variation in side pattern, ventral pattern, and geographic distance, none of these correlations remained significant when accounting for the effects of dorsal coloration, the variable with the highest *r* value in Mantel tests.

Within Cluster C, both our PCA analysis and our NGSadmix analysis indicated two distinct groups. Our NGSadmix analysis revealed two groups (*K* = 2 based on Evanno et al., 2005 method), one containing all *M. milotympanum *populations and one containing all *M. cf. milotympanum* populations (Figure 3). While our splitsTree analysis supported these two main groupings, there was a high degree of genetic admixture among populations in this cluster (Figure 2b) and populations were less well‐defined. The *F*_ST_ values between populations in this cluster were the lowest out of all pairwise population comparisons in this dataset, ranging from 0.14 to 0.25 (Figure 4). Despite the high degree of phenotypic variability in populations of this cluster, genetic variation was not correlated with any of the phenotypic traits that we quantified (Table 1). Genetic distance was only significantly correlated with geographic distance (*r* = 0.1038; *p*‐value = 0.018; Table 1). All three phenotypic traits, however, remained significantly correlated with geographic distance when accounting for genetic distance (dorsal coloration: *r* = 0.1353; *p*‐value = 0.009; side pattern: *r* = 0.2067; *p*‐value = 0.002; ventral pattern: *r* = 0.387; *p*‐value = 0.001). Despite this spatial segregation of phenotypes, we observed an exceptionally high degree of variation within populations in this group (Figure 5). For side and ventral pattern, in particular, within‐population variation was highest and discrete groupings were not apparent (Figure 5). Although there was less within‐population variation in dorsal chroma and hue, there was still not an obvious phenotypic split between *M. milotympanum* and *M. cf. milotympanum *populations (Figure 5). Overall, phenotypes in this group demonstrated much higher degrees of intrapopulational variation with less discrete groups emerging.

**Figure 6:**
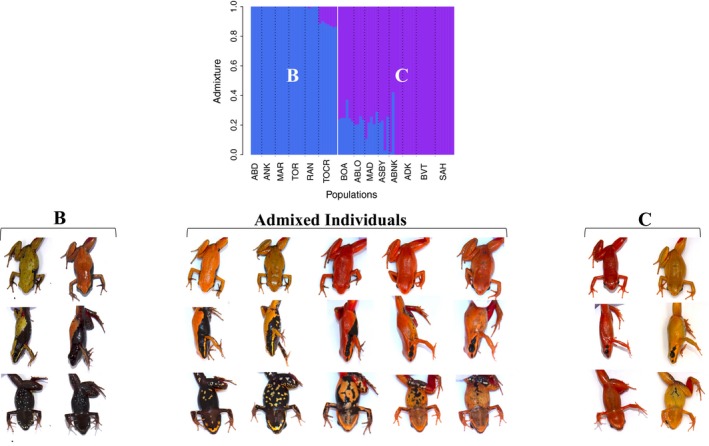
Genetic and phenotypic admixture among candidate hybrid populations. Top plot displays NGSadmix results for the subset of samples from Clusters B and C only. Dashed black lines separate geographic sampling locations, while the solid white line separates groups identified in NGSadmix analysis. Pictures under “B” and “C” headings show representative specimens from populations within each cluster (B or C), while pictures under “Admixed Individuals” show representative variation observed in individuals admixed between Clusters B and C. Individuals that are classified as *Mantella aurantiaca *are not pictured here under Cluster B. All variants displayed under “Admixed Individuals” come from a single population and demonstrate the spectrum of variation that occurs in candidate hybrid populations. Candidate hybrid populations displayed intermediate phenotypes primarily in terms of side and ventral patterning elements

One of the most interesting findings was evidence of possible introgression between *M. crocea/aurantiaca* and *M. milotympanum* populations. To further investigate this phenomenon, we repeated our NGSadmix and Mantel analysis on the subset of samples that included the admixed populations and the two clusters from which this admixture was drawn (Cluster B and Cluster C). Our NGSadmix results yielded a consistent signature of admixture in all *M. cf. milotympanum* populations that we sampled (Figure 6). In the wild, *M. cf. milotympanum *individuals demonstrate a side and ventral pattern that is intermediate between that observed in *M. crocea* and *M. milotympanum *(Figure 6). Despite the existence of these phenotypically and genotypically intermediate populations, genetic distance was not correlated with any of the phenotypic traits that we quantified, but was significantly correlated with geographic distance (*r* = 0.1391; *p*‐value = 0.050; Table 1). However, variation in dorsal coloration, side pattern, and ventral pattern was all significantly correlated with geographic distance even when controlling for the effects of genetic distance with partial Mantel tests (dorsal coloration: *r* = 0.2585; *p*‐value = 0.001; side pattern: *r* = 0.3873; *p*‐value = 0.001; ventral pattern: *r* = 0.3233; *p*‐value = 0.001). Notably, candidate hybrids, or *M. cf. milotympanum* individuals, demonstrated higher within‐population variation in patterning characteristics, and also displayed novel pattern phenotypes not present in either “parental” species (Figures 5 and 6). Within each candidate hybrid population, we observed a spectrum of pattern variants rather than discrete morphs.

## 4 DISCUSSION

Our investigation of color diversity and genetic structure in a complex of three closely related species of Malagasy poison frog revealed that the level of intraspecific color variation in this species complex has likely been overestimated, while the occurrence of distinct species has been underestimated. Although not in alignment with current species designations, we found evidence for several clear genetic clusters, each demonstrating a distinctive pattern of genetic and phenotypic variation, suggesting that a number of mechanisms are contributing to color evolution in this complex. Below, we discuss the relevance of our findings for taxonomy, conservation, and the evolutionary processes contributing to phenotypic variation in aposematic organisms. Additionally, we consider the implications of our findings for characterizing morphs in aposematic systems.

### 4.1 Taxonomic resolution and implications for conservation

Populations within this complex were highly structured at relatively small spatial scales, with distinct genetic groups emerging. Unexpectedly, genetic clusters identified in this study do not correspond to current species designations. Specifically, our results indicated that frog populations that have thus far been considered to be a green color variant of the *M. crocea* species form their own genetic cluster (Cluster A) and are in fact distinct from the other *M. crocea* populations included in this study (Figures 2 and 3). On the basis of this work, we suggest that what has previously been considered to be a green morph of *M. crocea* likely constitutes a new species, and we recommend further investigation of morphology, acoustics and behavior to delineate species boundaries with more certainty. The remaining (non‐green) *M. crocea* populations form a distinct cluster with the two *M. aurantiaca* populations (Cluster B; Figures 2 and 3). Although previous work has found evidence of haplotype sharing between *M. crocea* and *M. aurantiaca* (Chiari et al., 2004; Vences et al., 2004), it is surprising that populations of these two species are identified as a cohesive cluster in our analysis, as they differ greatly in color, pattern and body shape. Our work also demonstrates the validity of the *M. milotympanum* species (Cluster C; Figures 2 and 3). Previous studies have hypothesized *M. milotympanum* to be a color variant of *M. crocea* (Chiari et al., 2004; Vences et al., 2004), but our results demonstrate that *M. milotympanum* is genetically distinct from the clusters that contain what is currently called *M. crocea* (Clusters A and B).

Finally, we identified candidate hybrid populations (genetically admixed between Clusters B and C) that displayed intermediate genotypes and pattern phenotypes (Figures 3 and 6). Because earlier studies have hypothesized that *M. milotympanum* and *M. crocea* are conspecific, these intermediates were referred to as *M. cf. milotympanum* in the literature and assumed to be another phenotypic variant (Chiari et al., 2004; Vences et al., 2004). However, we demonstrate that *M. milotympanum *is its own distinct genetic entity, and likely experienced admixture with *M. crocea *at some point in the past. Although *M. milotympanum* and *M. crocea* do not currently live in sympatry to our knowledge, it is certainly feasible that these species either lived sympatrically in the past or have experienced secondary contact with each other, given the strikingly high degree of deforestation in Madagascar and the highly fragmented nature of remaining *Mantella* populations.

The taxonomic resolution provided by our study has significant consequences for conservation efforts, given that *M. aurantiaca* and *M. milotympanum* are currently classified as critically endangered, while *M. crocea* is classified as vulnerable (Andreone et al., 2005). Overall, populations were highly genetically structured with substantial phenotypic variation, indicating that there may be a number of distinct units to consider in management efforts. More importantly, genetic differentiation was not consistently correlated with phenotypic variation, emphasizing the importance of integrating both phenotypic and genetic information in prioritizing units for conservation. Based on our findings, we offer several recommendations for future management efforts. Because *M. milotympanum* represents a distinct species and is not a color morph of *M. crocea*, its status as critically endangered warrants high conservation priority. Future conservation endeavors will need to address the existence of populations admixed between *M. crocea* and *M. milotympanum*, and to determine where these populations fit in a conservation plan. The conservation status of green *M. crocea *populations should be reassessed, as these populations likely constitute a new species rather than a color morph of an already described species. Finally, within each major genetic cluster, our results suggest that there may be two distinct species or subspecies (Figure 3). We recommend that these subclusters be considered as distinct units for Cluster A (the green *M. crocea* cluster, where genetic differentiation is exceptionally high) and Cluster B (the *M. crocea‐M. aurantiaca* cluster, where phenotypic differentiation is exceptionally high). Due to the highly isolated and fragmented nature of *Mantella *populations, further intensive survey efforts will be important to identify any additional extant populations.

Overall, while it is premature to delineate new species on the basis of our analyses—especially considering the significant conservation implications in this system—our study confirms that there are at least three genetically distinct groups that do not correspond to current species descriptions. At the least, it seems clear that Cluster A (composed of green “*M. crocea*”) should be considered a distinct entity and prioritized given its extremely limited distribution (i.e., known from only two isolated locations). Due to the likely hybridization that we detected between Clusters B and C, and the fine‐scale population structure that we observed within each cluster, the status of Clusters B and C is less clear. Rather than revising taxonomy prematurely, we recommend additional studies on gene flow and migration, characteristics of frog calls, and mating behavior before species boundaries can be clarified with any certainty.

### 4.2 Divergent patterns of genetic and phenotypic diversity

Our findings suggest that a variety of processes at different spatial and genetic scales are likely contributing to differentiation in this Malagasy poison frog complex. Within major genetic clusters, we found evidence of highly regionalized patterns of phenotypic and genetic diversity (Figure 3). In our regression analysis, while genetic distance was most highly correlated with geographic distance for Clusters A and C, dorsal coloration was most highly correlated with genetic distance within Cluster B (Table 1). We also identified likely *M. crocea*‐*M. milotympanum* hybrid populations (populations genetically admixed between Clusters B and C) that displayed intermediate genotypes and novel pattern phenotypes (with especially high within‐population pattern variation; Figures 3, 5 and 6). Hybridization has been hypothesized to be an important mechanism of generating novel phenotypes in other poison frog systems (Medina, Wang, Salazar, & Amézquita, 2013) and may play a similar role here. Based on our findings, patterns of color evolution are likely influenced by variable patterns of drift, selection, and hybridization across major genetic groups in this Malagasy poison frog complex. Regional diversity in patterns of genetic and phenotypic variation has been found in other frog systems, including the red‐eyed treefrog *Agalychnis callidryas*, and lends support to the hypothesis that modes of diversification can vary substantially at relatively small spatial scales (Robertson & Zamudio, 2009).

While studies of phenotypic variation in Malagasy poison frogs are extremely limited, research on aposematic signal evolution in Neotropical poison frogs suggests that both natural and sexual selection likely contribute to phenotypic diversity (e.g., Reynolds & Fitzpatrick, 2007; Noonan & Comeault, 2009; Cummings & Crothers, 2013; Yang, Richards‐Zawacki, Devar, & Dugas, 2016). Previous work in *Oophaga pumilio* has demonstrated that male dorsal coloration is an important component of female mating preferences (Maan & Cummings, 2008, 2009) and that dorsal brightness is an important signal in male‐male competition (Crothers, Gering, & Cummings, 2011), while also confirming that conspicuous coloration is an honest signal to predators (Maan & Cummings, 2011). In fact, studies within this system have even suggested that natural and sexual selection may operate on different aspects of frog coloration, as variation in male brightness, an important component of assortative mating and male–male interactions, is not visible to avian predators (Crothers & Cummings, 2013). Additionally, there is growing evidence that pattern traits in poison frogs may also be under selection (Barnett, Michalis, Scott‐Samuel, & Cuthill, 2018; Rojas & Endler, 2013; Wollenberg, Lötters, Mora‐Ferrer, & Veith, 2008). Studies of intrapopulation pattern variation in the poison frog *Dendrobates tinctorius* have demonstrated that certain pattern traits are correlated with movement behavior, suggesting that patterning elements may also be important in determining how aposematic organisms are perceived by predators (Rojas, Devillechabrolle, & Endler, 2014). In fact, recent work in *D. tinctorius* indicates that pattern and color function as an aposematic signal to predators at close range, but are perceived as cryptic when viewed from longer distances (Barnett et al., 2018). Future work on phenotypic diversity in Malagasy poison frogs should draw on this body of literature and consider the relative roles of natural and sexual selection in shaping phenotypes, as well as the relative contributions of color and pattern to predator avoidance.

While Malagasy and Neotropical poison frogs demonstrate interesting parallels with each other, unique characteristics of the *Mantella *system render it particularly interesting from an evolutionary perspective. For example, while studies indicate that birds are particularly important predators of Neotropical poison frogs (Maan & Cummings, 2011; Saporito, Zuercher, Roberts, Gerow, & Donnelly, 2007), the only published instances of predation in Malagasy poison frogs were by snakes (*Thamnosophis* sp. in *M. aurantiaca*, and *Acrantophis madagascariensis* and *Compsophis laphystius* in *Mantella laevigata*) and lizards (*Zonosaurus *sp. in *M. aurantiaca* and *Zonosaurus madagascariensis* in *M. laevigata*) (Heying, 2001a; Hutter, Andriampenomanana, Razafindraibe, Rakotoarison, & Scherz, 2018; Jovanovic, Vences, Safarek, Rabemananjara, & Dolch, 2009). Although the extent to which snakes and lizards utilize visual signals—especially in relation to olfactory cues—in predation is not fully understood (Maan & Cummings, 2011; Willink, García‐Rodríguez, Bolaños, & Pröhl, 2014), lizards have been found to select for lower conspicuousness in the poison frog *Oophaga granulifera* (Willink et al., 2014). Consequently, there is reason to believe that selective pressures resulting from predation—presumably a major driving force in shaping phenotypic variation in aposematic organisms—may be substantially different for Malagasy poison frogs. Thus, the Malagasy poison frogs represent an important unique and comparative system for developing generality about the selective factors influencing color evolution. To understand the processes generating the interesting and variable patterns of phenotypic diversity, there is a pronounced need for research on the ecology and life history of these frogs. Fundamental research on *Mantella *predation, migration, mating behavior, diet, toxicity, and habitat quantification will be essential in formulating explicit hypotheses regarding color evolution.

### 4.3 Broader implications for defining species and morphs in phenotypically diverse aposematic organisms

Our results demonstrate that characterization of morphs based solely on observed phenotypic variation, especially when genetic structure is unresolved, can lead to an overestimation of the degree of polymorphism and/or polytypism that occurs in aposematic systems. Our findings are consistent with other studies in Neotropical poison frogs where sophisticated genetic datasets, utilized either in isolation or paired with morphological and ecological data, have revealed that a single species likely includes several distinct lineages (Posso‐Terranova & Andrés, 2018; Roland et al., 2017). Thus, overestimation of intraspecific variation and underestimation of species diversity in phenotypically diverse lineages may be more widespread than previously appreciated. In addition to potentially leading to erroneous evolutionary inferences, underestimating species diversity can also have important conservation implications if newly identified lineages are highly restricted in their distribution and/or exist outside of protected areas (Posso‐Terranova & Andrés, 2018), as is the case for this complex of Malagasy poison frogs.

In this study, although our high‐resolution genomic dataset clarified species boundaries at the highest level, the way in which fine‐scale population structure is interpreted has significant implications for how hypotheses of color evolution are framed in this system. Within Cluster B, for example, if *M. aurantiaca* and *M. crocea* are considered to be color morphs of the same species, then one interpretation of our results is that differences in dorsal coloration are driving reproductive isolation and may be a potential mechanism for incipient speciation in this group, as hypothesized in another polymorphic poison frog complex (Wang & Summers, 2010). If considered to be separate species, however, then the dramatic phenotypic differences that coincide with genetic variation may serve to reinforce species recognition and to prevent hybridization. Our study demonstrates that even when genetic structure is well understood, distinguishing between species, subspecies and morphs is not always a straightforward process and may require additional information on behavior, acoustics and mating preferences, among other traits.

For aposematic organisms presumed to be polymorphic or polytypic, our results emphasize that more thorough deliberation is necessary not only in the delineation of species but also in the characterization of morphs. Refining our understanding of what constitutes a morph, and when species or populations are polymorphic and/or polytypic, may be a necessary first step in this regard. Below, we highlight three conceptual areas identified on the basis of our findings that merit more careful consideration in the identification and description of phenotypic morphs.

Although phenotypes are multifaceted, morphs are often defined on the basis of one charismatic trait. Consequently, designating variants of one phenotypic axis as morphs is premature and fails to account for variation across multiple traits. In our study, patterns of variation in coloration and pattern traits were not always concordant; interpopulation differences in dorsal coloration were much more discrete than differences in pattern, although both varied among populations (Figure 5). Within most candidate hybrid populations, dorsal coloration was relatively uniform, but patterning elements varied almost continuously along a spectrum (Figures 5 and 6). These findings highlight the difficulties of characterizing distinct morphs when there is variation along multiple, potentially correlated phenotypic axes. Similar findings have been reported in other poison frog species, where continuous pattern variation was observed within populations (Rojas & Endler, 2013). In such instances, explicitly describing and quantifying variation—whether it is continuous or discrete—in multiple phenotypic traits is essential. We recommend caution in designating discrete morphs when there may be continuous phenotypic variation, especially along multiple phenotypic axes.

In aposematic organisms, designation of morphs is often based on human‐observed phenotypic variation. This raises the question: is there really that much diversity in aposematic signals, when considered from the relevant predator and conspecific visual perspectives? In our study, although populations varied in terms of dorsal chroma and hue, dorsal luminance was largely similar across most populations (Figure 5). Interpreted in light of evidence suggesting that high luminance contrast can serve as an effective warning signal to predators (Prudic, Skemp, & Papaj, 2007), and that dorsal brightness is an important component of conspecific signaling in poison frogs (Crothers et al., 2011; Maan & Cummings, 2009), biologically meaningful color diversity in this system may be much less than expected. Although we observed high degrees of pattern variation on the side and ventral surfaces of frogs in our study (Figures 5 and 6), the role of this variation is unknown. Recent work in poison frogs linking patterning elements to movement behavior and detectability by predators (Barnett et al., 2018; Rojas, Devillechabrolle et al., 2014) indicates that pattern variation may be equally, or even more, biologically relevant than color variation. Further, evidence that the detectability of different color pattern variants is influenced by the existing light environment (Rojas, Rautiala, & Mappes, 2014) underscores the importance of incorporating information on both predator sensory abilities and ambient lighting conditions into the characterization of phenotypic variation. Moving forward, it is important to determine which aspects of coloration and/or pattern are perceived by predators and conspecifics. Then, morphs should be defined on the basis ofthese biologically relevant visual perspectives to accurately capture the degree of diversity in aposematic warning signals.

Finally, our study underscores the need for a reassessment of how morphs are characterized in evolutionary biology. Determining whether color variants represent different species or morphs requires, at the least, sophisticated genomics datasets and an explicit integration of multidimensional phenotypic data. At a broad conceptual scale, our results clearly indicate that levels of intraspecific color variation in this complex of Malagasy poison frogs have been overestimated. At the same time, our results underscore the difficulty in delineating species and morphs at a fine scale in highly complex systems, particularly when genetic and phenotypic breaks are not predictably correlated (Figure 3). Even with robust genomic data, defining morphs can still be challenging, especially when species boundaries are not straightforward. Polymorphism and polytypism cannot be studied if it is not clear whether discrete morphs exist and whether observed variation is within populations, among populations, or between species.

So, what is a morph? Evolutionary biologists often use the term “morph” without a formal conceptual or operational definition. The research community has thought carefully over decades about how to define and delineate species (e.g., Mayr, 1942; Wiley, 1978; Mallet, 1995; Sites & Marshall, 2004; de Queiroz, 2007). The same attention has not been paid to the morph concept (but see Teasdale, Stevens, & Stuart‐Fox, 2013). Not only do we need a more standardized definition of morph but also operational criteria for delineating morphs when there is phenotypic variation along multiple axes and/or when species boundaries are unclear or dynamic through time.

Our purpose here is not to provide a revised definition of “morph” but rather to highlight the importance of contextualizing phenotypic variation appropriately. When a novel phenotypic variant is discovered, rather than prematurely being described as a new species or morph, it should serve as a launching point for comprehensive studies that integrate phenotypic information with genomic datasets (and, ideally, information on acoustics and behavior). Describing novel phenotypic variants within a species should require—at the least—a high‐resolution genetic dataset to confirm intraspecific relationships. In addition, rather than characterizing human‐observed variants as morphs, we recommend that researchers specify the facet of phenotype measured, the sensory perspective used to quantify phenotype, and the level of biological organization where variation is observed. The way we define and delineate morphs is relevant in many systems, but carries particular significance in aposematic organisms, where novel color and pattern variants are regularly found. If we are overestimating intraspecific color variation and underestimating species diversity—as was found in this study—the classification of populations and species as polymorphic or polytypic may need to be reevaluated. Ultimately, it is time to give as much consideration to the conceptual and operational morph delineation as has been given to species.

## CONFLICT OF INTEREST

None declared.

## AUTHOR CONTRIBUTIONS

KK designed the research with input from EBR. KK performed the field work and lab work. KK and KB analyzed the data. KK and EBR wrote the paper with contributions from KB.

## Data Availability

RADseq data will be deposited in the NCBI Sequence Read Archive (SRA).
